# Next-generation models for lymphoid malignancies: the rise of 3D culture systems in translational hematology

**DOI:** 10.1038/s41416-026-03487-x

**Published:** 2026-06-03

**Authors:** Narimène Houmera, Laurent Genestier, Sarah Huet

**Affiliations:** 1 Team Lymphoma Immuno-Biology, Centre International de Recherche en Infectiologie, Lyon, France; 2 Institut des Sciences Pharmaceutiques et Biologiques, Université Claude Bernard Lyon 1, Lyon, France; 3 Service d’hématologie biologique, Hôpital Lyon Sud, Hospices Civils de Lyon, Lyon, France

**Keywords:** Predictive markers, Haematological cancer, Cancer models

## Abstract

Traditional models used to study lymphoid malignancies, such as 2D cell cultures and murine systems, have significantly advanced our understanding of tumour biology and drug development. However, their limited capacity to recapitulate the tumour microenvironment and 3D anatomical structure restricts their translational relevance. In response to these challenges, three-dimensional (3D) culture systems have recently emerged as promising platforms to more accurately replicate the architecture and biological complexity of lymphoid tissues. A variety of 3D models have been developed, ranging from simple spheroids to advanced organ-on-chip technologies that allow for continuous perfusion and precise modulation of microenvironmental parameters. Current optimisation efforts aim to enhance these systems’ ability to sustain lymphoma cell viability and mimic key in vivo features such as stromal integration, spatial organisation, and biomechanical cues. This review provides an overview of the current 3D models used to investigate mature lymphoid malignancies, with a particular focus on chronic lymphocytic leukaemia and lymphomas. We discuss their relevance, strengths, and limitations, especially in the context of therapeutic screening and the advancement of personalised treatment approaches.

## Background

Mature lymphoid malignancies, such as chronic lymphocytic leukaemia (CLL), multiple myeloma, and lymphomas, represent a heterogeneous group of cancers with increasing incidence over the past decades [1, 2]. Despite therapeutic advances, most remain incurable, characterised by recurrent relapses. Traditional two-dimensional (2D) cell culture models have long supported drug discovery due to their simplicity and reproducibility, but they inadequately mimic the complexity of primary tumours. Cancer cell lines often undergo genotypic and phenotypic drift during long-term culture, losing key tumour-specific traits [3, 4]. Conventional in vitro culture of primary tumour cell offer greater fidelity but face challenges, notably the poor survival of certain lymphoma subtypes or CLL cells ex vivo, largely due to the absence of their native microenvironment [5–7**]**.

The tumour microenvironment (TME), composed of stromal cells, cytokines, and extracellular matrix (ECM) components, plays a central role in supporting malignant cell survival and progression. To better replicate this niche, co-culture systems incorporating stromal and helper-like cells have been developed, alongside cytokine supplementation—e.g. interleukin (IL)-4, IL15, IL21, B-cell-activating factor (BAFF), CD40L, CCL2… For instance, co-culturing CLL cells with stromal cells—such as bone marrow stromal cells, blood-derived nurse-like cells, and extramedullary stromal cells of mesenchymal origin—rescues CLL cells from apoptosis [8, 9]. This protective effect appears to be mediated by interactions between chemokines and their receptors on the surface of CLL cells, with stromal cell-derived factor 1 (SDF-1) playing a key role in preventing apoptosis. Similarly, co-culturing primary follicular lymphoma (FL) cells with follicular dendritic cell-like cells drastically reduces apoptosis [6]. The interaction between B cells and T follicular helper cells, particularly through the CD40–CD40L pathway, is critical for B-cell survival. Accordingly, various CD40L-expressing cell lines are commonly used in co-culture systems for germinal centre (GC)-derived tumour cells. However, these approaches only partially restore tumour-supportive interactions, in part because supporting cells are not of intratumoral origin and because 2D cultures lack the spatial architecture and ECM of in vivo tumours. Consequently, recreating a physiologically relevant microenvironment in vitro remains a key challenge in modelling lymphoid malignancies.

Murine models (extensively reviewed elsewhere [10–12]), while more physiologically relevant than 2D systems regarding tumour biology, immune interactions and therapeutic responses, also face significant limitations. These include inadequate representation of tumour heterogeneity, potential cross-reactivity between murine and human immune targets during immunotherapy testing, and extended engraftment times, that can range from 10 to over 15 months for lymphomas.

Moreover, despite the development of a wide range of targeted therapies for haematological malignancies in recent years, only about 5% of newly tested molecules achieve clinical approval [13], including a 50% failure rate in phase III trials [14]. This high failure rate is often attributed to the limitations of preclinical models, which do not fully replicate the complexity of in vivo tumours or the interactions between tumour cells and their microenvironment [15].

To address these gaps, there has been growing interest in developing 3D culture models. These innovative systems aim to recapitulate the complex architecture and microenvironment of tissues like the bone marrow (BM) and lymph nodes while remaining in vitro. The objective of this review is to provide a comprehensive exploration of the three-dimensional (3D) systems used to study mature lymphoid malignancies, focusing on lymphomas and CLL that share many physiopathological features, but excluding multiple myeloma (reviewed elsewhere [16]). We will examine the ability of these models to accurately simulate disease conditions and their utility in therapeutic screening.

## Advances in 3D models of mature lymphoid tumours

Given the complexity of these diseases and the reliance of tumour cells on interactions with the microenvironment, models need to closely mimic in vivo conditions to be truly representative. The ability to maintain primary cells from patients in these systems is crucial, as it recapitulates the heterogeneity and complexity of real-world tumours, allowing for a more accurate assessment of treatment efficacy. Most 3D culture models have been developed to recapitulate secondary lymphoid organs, where lymphomas arise. However, specialised systems based on scaffolds mimicking trabecular bone have also been described to study CLL and diffuse large B-cell lymphoma (DLBCL) with BM involvement. The following paragraphs will delve into the models that have been developed to address these issues, highlighting their respective advantages and limitations (Table 1).

**Table 1:** Advantages and limitations of 3D culture systems.

Advantages	Limitations
**Scaffold-free models**	
General	
• Relatively low cost and easy to implement in standard laboratories. • High control over initial cell seeding density and spheroid uniformity.	• Static culture conditions, with limited control of gradients and no active perfusion. • Poor diffusion of oxygen, nutrients, and drugs within the inner core, leading to hypoxia and necrotic areas depending on spheroid size. • Lack of compartmentalisation and vascular components • Lack of stromal or immune components unless these are intentionally co-cultured.
Hanging-drop spheroids	
	• Spheroids are difficult to handle outside the seeding platform and often require manual transfer into agarose- or ULA-coated plates. • Labour-intensive and poorly suited to large-scale or fully automated workflows; droplet coalescence can impair reproducibility.
ULA (ultra-low attachment) plates	
• User-friendly, compatible with multi-well plate formats; suitable for medium- to high-throughput drug screening. • Reliable formation of single, round spheroids per well with good reproducibility in size and morphology.	
Microencapsulation	
• Provides a protective 3D micro-niche, allowing long-term culture and co-culture (e.g. stromal and immune cells). • Amenable to high-throughput formats; multiple, highly reproducible replicates can be generated in parallel.	• Requires dedicated microfluidic or encapsulation devices and technical expertise. • Limited imaging and downstream recovery of viable cells from capsules
**Hydrogel-based scaffolds**	
• Relatively low cost and widely available; compatible with various matrix compositions (e.g. collagen, Matrigel, synthetic hydrogels). • The hydrogel facilitates cell inclusion and sample handling; mechanical stiffness and viscoelasticity can be finely tuned • Functionalization with peptides or ECM components enhances cell adhesion, survival, and differentiation, and allows modelling of specific microenvironmental cues.	• Static culture unless combined with perfusion systems; diffusion gradients can lead to heterogeneous exposure to drugs and cytokines. • Technical variability linked to manual embedding and polymerisation • Limited control over precise 3D architecture and compartmentalisation • Optical density of some hydrogels complicates high-resolution imaging.
**Inclusion of primary tissue fragments (explants, organoids)**	
• Preserves native 3D architecture, stromal composition, and extracellular matrix, maintaining key cell–cell and cell–matrix interactions. • High biological relevance; closely reflects inter- and intra-patient heterogeneity, supporting personalised drug testing.	• Static culture, with diffusion limitations and progressive tissue degradation over time. • Reproducibility is constrained by availability, quantity, and quality of primary tissue; limited scalability and standardisation.
**Organ-on-chip/microfluidic systems**	
• Dynamic culture with controlled perfusion of nutrients, oxygen, cytokines, and drugs, allowing definition of physiological gradients. • Precise control over 3D compartmentalisation and microchannel geometry, enabling modelling of cell–endothelium interactions, vascular niches, and tissue–tissue interfaces. • Ability to impose controlled fluidic shear stress and mechanical cues	• More expensive and technically demanding; requires specialised equipment, microfabrication, and expertise in microfluidics. • Lower throughput than plate-based systems
**Hard material-based, preformed scaffolds (e.g. decellularized bone, synthetic porous matrices)**	
General	
• Defined control over 3D compartmentalisation and mechanical properties, enabling modelling of bone marrow niches and spatially organised lymphoid structures. • Highly reproducible scaffold geometry and pore structure; often available as off-the-shelf products (for synthetic matrices).	• More expensive than simple scaffold-free approaches; requires specific handling and sometimes bioreactor integration. • In the case of decellularized human scaffolds, limited by donor tissue availability and batch variability. • Limited capacity for remodelling in very stiff or inert materials; may not fully recapitulate dynamic ECM turnover. • Static unless transferred into bioreactors
Hard scaffolds with bioreactor (e.g. rotating or perfusion bioreactors)	
• Transfer to rotating or perfusion bioreactors allows control of mechanical forces and fluid flow, generating drug and oxygen distribution patterns closer to in vivo conditions. • Particularly relevant to study cell trafficking between distinct compartments (e.g. CLL recirculation between blood, node, and bone marrow-like niches).	• Requires dedicated, often expensive bioreactor equipment and specific expertise • Lower throughput and more complex experimental design compared with simple plate-based 3D cultures.

We will adopt a previously described classification [17] dividing 3D culture methodologies into two main groups: scaffold-free and scaffold-based methods. Scaffold-free models form cellular aggregates without external matrix support, while scaffold-based models seed or include cells into scaffolds providing biophysical support for growth and aggregation, but also actively supporting cell growth, proliferation, and biological activities.

Beyond this general classification, nomenclature can vary across studies. This is the case for the term ‘organoid’, so we wish to clarify this definition at the outset of this manuscript. The term ‘organoid’ originally refers to a 3D cell culture system that mimics the structure and function of a specific organ or tissue. Organoids are typically derived from pluripotent stem cells or from isolated organ progenitors, cultured within biomaterials like synthetic polymer (e.g. polyethylene glycol), natural materials (e.g. collagen, alginate), decellularized native tissues [15] or in transwells [18]. When supplemented with growth and differentiation factors, these cells can undergo differentiation and self-organisation into complex, multi-cellular structures resembling native organs or tissues [19]. Although not technically organoids, some models—both scaffold-free and scaffold-based—use this terminology when aiming to replicate the anatomical and functional complexity of the GC.

### Scaffold-free models

Spheroids are formed from non-adherent cells that aggregate into a round structure, without the use of an external matrix as a biophysical support. Spheroid models have been instrumental in investigating tumour-stroma interactions, drug resistance and immune responses in B-cell lymphomas such as mantle cell lymphoma (MCL), CLL, DLBCL and FL, as described below.

#### Hanging drop method

**Fig. 1:**
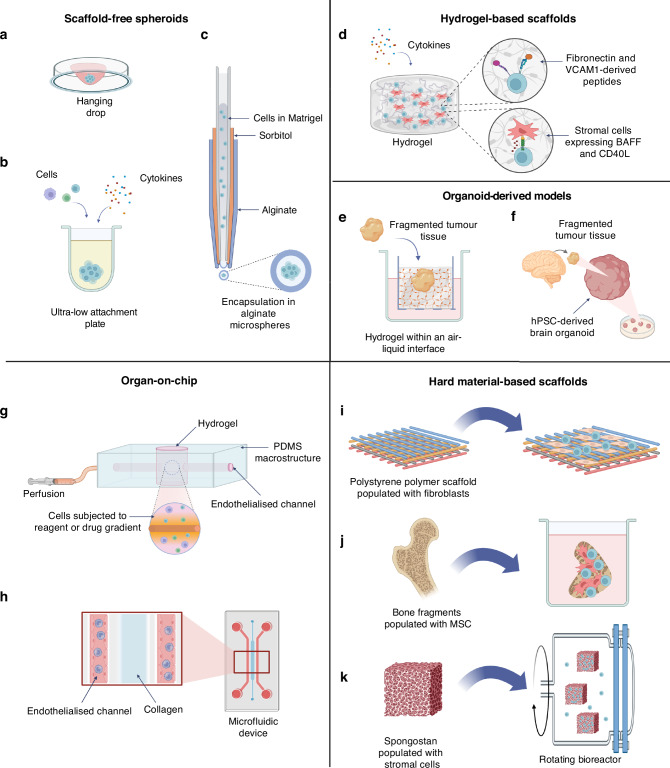
Overview of the different 3D culture systems engineered for mature lymphoid neoplasms. Speroids result of the aggregation of cells that aggregate into a round structure—**a** the hanging drop method, **b** culture in ultra-low attachment plates or **c** encapsulation of cells within alginate microspheres. In hydrogel-based scaffolds cells are embedded into polymer networks providing biophysical support for structural organisation and cellular activities—**d** ECM-mimicking hydrogel functionalized with integrin-binding peptides and populated with CD40L-expressing stromal cells to recreate tumour microenvironment; Organoid-derived models are designed to recreate or keep the original anatomical structure—**e** primary tumour biopsy from lymph node embedded within hydrogel in an air-liquid system; **f** primary tumour biopsy from central nervous system lymphoma embedded within brain organoids with Matrigel. Organ-on-chip models allow the modelling of interactions between tumour and endothelial cells—**g** hydrogel containing lymphoma and immune cells, embedded within a polydimethylsiloxane (PDMS) macrostructure with a perfusable blood vessel; **h** 3D microfluidic chip model consisting of a central collagen hydrogel channel (perivascular niche), flanked by two lateral channels mimicking blood vessels. Hard material-based scaffolds rely on on a rigid structure populated with stromal cells—**i** polystyrene scaffold; **j** bone fragments; **k** biomaterial such as Spongostan, populated with stromal cells. Created in BioRender @Sarah_Huet

Early models employed the hanging drop method, which involves placing a small droplet of cell suspension on the lid of a culture plate that is then inverted, and the droplets remain attached to the lid due to surface tension [20] (Fig. 1a). This technique relies on gravity-driven self-assembly of cells to form multicellular spheroids. The Multicellular Aggregates of Lymphoma Cells (MALC) model has been developed using this method on transformed FL cell lines [21–24]. This model produces spheroids that can be cultured for up to three weeks, replicating key features of FL tumour architecture, such as enhanced deposition of type I collagen, fibronectin, and laminin, and hypoxic conditions mediated by the Hypoxia Inducible Factor 1 Subunit Alpha (HIF1α) [22, 23]. Interestingly, chemotherapies like doxorubicin and bendamustine showed reduced efficacy in spheroids compared to 2D culture, due to the presence of quiescent cells and downregulated topoisomerase II-α. On the contrary, anti-CD20 monoclonal antibodies were more effective in 3D cultures than in 2D systems. These findings suggest that conformational changes may induce alterations in key intracellular signalling pathways. Combined with the increased proximity of immune cells in the 3D structure, this leads to enhanced treatment efficacy [21, 22]. The MALC model also highlighted immune evasion mechanisms in FL, as co-culturing FL cells with natural killer (NK) cells derived from healthy donors revealed significantly lower lysis rates in spheroids compared to 2D cultures. However, the addition of rituximab or obinutuzumab restored cytotoxicity, demonstrating the ability of monoclonal antibodies to penetrate the 3D structure and activate NK cells and γδ T cells [22–24].

#### Ultra-low attachment plates

Spheroids obtained with the hanging-drop method are difficult to handle outside the seeding platform and necessitate manual transfer of neoaggregates into agarose-precoated wells. This technique has further evolved with the use of ultra-low attachment (ULA) plates, in which spheroids float freely within the culture media (Fig. 1b), allowing 3D imaging to characterise morphological properties of the spheroids [25].

Duś-Szachniewicz et al. [26] addressed the question of cell-adhesion mediated drug resistance by producing hybrid lymphoma/stromal spheroids in a commercially available hydrogel microwell system. Co-culturing of DLBCL-derived cell lines (Ri-1 and RAJI) with mesenchymal stromal cells (HS-5 fibroblasts) enhanced cell aggregation within spheroids and cell survival, extending viability at 14 days of culture (but not at 7 days). Notably, after treatment with the Bruton tyrosine kinase inhibitor ibrutinib, the decrease of spheroid diameter and cell viability was more pronounced in mono-culture than in the co-culture condition, further highlighting the role of stromal cells in drug resistance.

Adapted from the previous MALC system, the ‘FL patient-derived lymphoma spheroids’ (FL-PDLS) model has been recently adapted to primary FL cells. Cultured in ULA plates with a cytokine cocktail comprising IL2, IL4, IL15 and CD40L, primary lymphoma cells formed spheroids that retained their tumour immune microenvironment, including immune cell populations such as CD4^+^ and CD8^+^ T cells and NK cells [27]. The FL-PDLS model was further adapted by the addition of purified monocytes from healthy donors [28]. This model was able to maintain FL cell viability over seven days, although the spheroids revealed an altered gene expression profile compared to primary biopsies, with a notable overexpression of survival and proliferation signatures [27]. Interestingly, the T cell compartment in FL-PDLS recapitulated features of the native FL microenvironment with different T cell subsets encompassing a spectrum of activation/exhaustion phenotypes and preserving the main T cell subpopulations. The authors evaluated the in vitro response to the first-line treatment (rituximab, cyclophosphamide, doxorubicin, vincristine, prednisolone) and different immune checkpoint inhibitors. Antibodies targeting the inducible T cell costimulatory (ICOS), the T-cell immunoglobulin mucin receptor 3 (TIM3), the lymphocyte activating gene 3 protein (LAG3), or the T-cell immunoreceptor with immunoglobulin and ITIM domains (TIGIT), resulted in limited and highly heterogeneous responses, whereas nivolumab—inhibitor of the programmed cell death protein 1—was the most effective immune checkpoint inhibitor that also increased rituximab activity [28]. This model has more recently been used to assess ex vivo responses to glofitamab in relapsed or refractory FL and DLBCL [29].

The second spheroid model adapted to primary FL cells was developed by Kastenschmidt et al. [30]. The 'patient-derived lymphoma organoids' (PDLO) model refers to a spheroid system that was first used to decipher the cellular dynamics underlying responses to influenza vaccine [31]. The model successfully maintained cellular viability of primary FL cells for up to 21 days, while preserving the original composition of tumour and immune cells as assessed by gene-expression profiling, mutational profiling and B/T cell receptor dynamics at different culture time points. PDLO treated with a CD19-specific T cell engager showed a significant decrease in the number of lymphoma cells, whereas CD8^+^ and CD4^+^ T cell populations showed evidence of activation. Although effective, treatment with a CD20-specific T cell engager did not induce the same magnitude of changes in lymphoma cells and in T cells.

Araujo et al. [32] optimised a spheroid model for MCL, incorporating signals from T follicular helper cells and follicular dendritic cells (CD40L, IL4 and BAFF) and monocytes from peripheral blood mononuclear cells (PBMC), which improved the viability and proliferation of primary MCL cells. Moreover, PDLS culture induced the differentiation of monocytes into M2 macrophages expressing CD206 and CCL22, as well as an exhaustion phenotype in T cells, thus recapitulating the biological features of MCL. The major advantage of this model lies in its ability to predict the in vivo responses of patients to ibrutinib after culturing samples from patients with known clinical follow-up regarding their therapy. In vitro, samples from responders showed decreased B-cell viability upon the addition of ibrutinib, while samples from non-responders benefited from combining ibrutinib with nivolumab. This combination increased interferon γ and granzyme B levels, reflecting enhanced cytotoxicity from immune cells.

In CLL, Haselager et al. [33] developed a 3D culture model using PBMC from CLL patients stimulated with an IL-2/IL-15/IL-21/CpG cocktail. In this model, 3D culture promoted greater expansion and long-term proliferation of CLL cells compared to 2D culture, with spheroids growing up to 4 × 10⁶ µm². This expansion was dependent on the presence of T cells, possibly reflecting improved interactions between CLL cells and surrounding T cells in the 3D environment, as spheroids began to develop follicle-like structures after 3–4 weeks of culture. Treatment with the Bruton tyrosine kinase inhibitor acalabrutinib or the B-cell lymphoma 2 inhibitor venetoclax reduced spheroid size and altered spheroid architecture. Interestingly, in two patients with paired baseline and refractory samples, the effect of acalabrutinib was diminished in the refractory samples, recapitulating in vitro the drug resistance observed in vivo. Thus, this 3D model enables the investigation of CLL cells within their natural protective niche and provides insights into the role of this environment in CLL drug resistance. More recently, it has been used to address specific questions regarding CLL pathophysiology and to suggest new therapeutic strategies, aiming to enhance the efficacy of targeted therapies and overcome resistance mechanisms [34, 35].

#### Microencapsulation

Lamaison et al. [36] adapted a method, initially developed for solid tumours [37, 38], where a microfluidic device encapsulates cells in alginate microspheres (Fig. 1c). Lymphoma B cells and tonsil stromal cells (TSC) were incorporated with Matrigel as ECM component within 216 μm-diameter microspheres and cells dynamically formed self-organised 3D spheroids inside the capsule. Using the DLBCL-derived SUDHL4 and HLY1 cell lines, microspheres seeded with as few as 18 cells supported cell proliferation. The early stages of spheroid growth were characterised by a high proliferation rate, while the post-confluent stage exhibited a significant decrease in cell proliferation concomitant with an increase in cell death after 8 days, although this was not associated with the formation of a dead cellular core, as observed in solid tumour spheroids [39]. Interestingly, cells from the DOHH2 cell line (derived from a patient with transformed FL) were not able to form spheroids in the absence of stromal cells and ECM [36]. Only in the presence of Matrigel, TSC anchored and spread onto ECM, leading to the formation of a ramified 3D network that subsequently supported DOHH2 cell growth, suggesting their requirement of a supportive microenvironment in this 3D context. This model also revealed enhanced resistance to doxorubicin in 3D cultures compared to 2D. However, diffusion limitations within the microspheres likely contributed to this resistance, underestimating the drug’s efficacy. Despite these challenges, primary FL cells were successfully maintained for up to 11 days, demonstrating the model’s potential for studying primary cells.

In conclusion, spheroid models offer a versatile platform for studying CLL and lymphomas and can be tuned by the addition of stromal cells, monocyte-derived macrophages and various cytokines, allowing for a closer approximation of the original tumour studied. Co-culture with stromal cells (able to produce ECM networks) helps creating a more cohesive spheroid. These methods have the advantages of cost-effectiveness, spheroid size control and produce spheroids that are stable over time, making them a valuable tool to study long-term effects of anticancer drugs [21, 40, 41]. However, features such as nutrient (e.g. glucose) and oxygen gradients, or ECM-driven biomechanical forces are only partially mimicked, and recreation of the native TME is inconsistently reproduced across studies. While most studies used lymphoma cell lines to establish a proof of principle, a few have addressed the challenge of culturing primary tumour cells (recapitulated in Table 2). Interestingly, several studies compared 2D and 3D culture conditions, with most demonstrating that direct or immune-mediated treatment responses differ based on culture dimensionality (2D vs. 3D). To our knowledge, the study by Araujo et al. is the only one that has successfully predicted patients’ clinical response through in vitro therapeutic screening [32]. Of note, a model based on Transwell culture inserts has also been described in a reactive context, where tonsillar B cells spontaneously reaggregate into clusters after seven days, mimicking GC organisation [42]. Although promising, this technique has so far not been adapted to lymphoma cell culture.

**Table 2:** Summary of 3D studies conducted on primary cells from patients with lymphoma or chronic lymphocytic leukaemia.

3D in vitro model	Reference	Pathology	Technical conditions	Number of patients	Cell viability	Therapeutic screening	Comparison 3D/2D on primary cells
**Spheroids**	Araujo-Ayala et al. [32]	Mantle lymphoma	Ultra-low attachment plates. 5 × 10^4^ primary cells (mostly from PB) and 1.25 × 10^4^ monocytes (4:1 ratio). Cytokines: IL4, BAFF, CD40L. Culture over 7 days.	*n* = 19	Viability of 20–95% depending on samples at d7.	Prediction of patient’s clinical response to ibrutinib.	No survival data in 2D. Transcriptomic profile closer to MCL primary samples in 3D than 2D.
	Faria et al. [27]	Follicular lymphoma	Ultra-low attachment plates. 2.5 × 10^4^ cells from the primary biopsy per spheroid. Cytokines: IL2, IL4, IL15, CD40L. Culture over 6 days.	*n* = 9	No viability data available but the volume of spheroids increased between d3 and d6.	Obinutuzumab and nivolumab decreased cell survival.	NA
	Dobaño-López et al. [28]	Follicular lymphoma	Ultra-low attachment plates. 5 × 10^4^ cells from the primary biopsy or PB and 1.25 × 10^4^ monocytes (4:1 ratio) per well. Cytokines: IL21, IL4, IL15, CD40L. Culture over 7-10 days.	*n* = 20	Viability of approx. 50% at d7. Proliferation increased by the addition of monocytes and cytokines.	Several immune checkpoint inhibitors tested. Rituximab, nivolumab and anti-galectin-9 antibodies induced B cell depletion in a subset of samples.	NA
	Kastenschmidt et al. [30]	Follicular lymphoma	Ultra-low attachment plates. 1.5 × 10^6^ cells from the primary biopsy per well. Culture over 21 days.	*n* = 12	Viability of 25–80% depending on samples, stable between d7 and d21.	Bispecific antibody CD3:CD19 showed a higher activity against lymphoma cells than CD3:CD20.	NA
	Lamaison et al. [36]	Follicular lymphoma	Encapsulation of FL cells in alginate microspheres in the presence of stromal cells (6:1 ratio) within matrigel (number of cells in each individual capsule not specified). Culture over 14 days.	*n* = 2	The number of cells per capsule was reduced by half between d3 and d14 (no survival data compared to d0).	Performed on cell lines only. Resistance to doxorubicin in 3D compared to 2D, due to impaired diffusion within spheroids when cells reach confluence.	Higher percentage of surviving primary FL cells at d11 in 3D compared to 2D.
	Haselager et al. [33]	Chronic lymphocytic leukaemia	Ultra-low attachment plates. 3 × 10^5^ total PBMC. IL-2, IL-15, IL-21, and the TLR9 agonist CpG or T cell stimulation using αCD3 and αCD28 antibodies, Culture up to 7 weeks.	*n* = 24	2-fold increase of CLL cells after 2 weeks.	Acalabrutinib and venetoclax decreased spheroid size. T-cell cytotoxicity assay with blinatumomab induced CLL cell lysis.	No proliferation of CLL cells in 2D. Higher blinatumomab-induced CLL cell lysis in 3D compared to 2D culture.
Hydrogel-based scaffolds	Lenti et al. [43]	Chronic lymphocytic leukaemia	Hanging drop method with type I collagen matrix. 4 × 10^5^ purified CLL cells and 2 × 10^4^ FRC cells (20:1 ratio). Culture over 4–10 days.	*n* = 7–11	Approx. 80%, stable between d4 and d10.	Venetoclax affected cell viability in 3D, whereas ibrutinib did not.	Similar survival rates between 2D and 3D when CLL cells cocultured with FRC. CLL:FRC ratio more stable in 3D. Higher apoptosis rates in 2D upon treatment with ibrutinib or venetoclax.
	Foxall et al. [5]	Diffuse large B cell lymphoma	Hanging drop method with type I collagen matrix. 10^6^ primary DLBCL cells in the presence of ADSC-derived stromal cells (100:1 ratio) and MDM (ratio up to 10:1) Culture over 8 days.	*n* = [1–8]^a^	Viability of 20-80% depending on samples, at d8.	Rituximab treatment did not induce a significant increase in phagocytosis or cell death.	Similar results regarding viability of DLBCL cells co-cultured with stromal cells. Increased phagocytosis with rituximab treatment in 2D.
	Shah et al. [46]	Diffuse large B cell lymphoma	Cell inclusion into a hydrogel matrix 0.8–2 × 10^5^ primary cells from PDX with 5 × 10^4^ stromal cells expressing CD40L. Culture over 4 days.	*n* = 8	No raw data regarding viability.	Exposure to MALT1 inhibitors induced a reduced cell death in the presence of CD40L-expressing stromal cells (n = 3 PDX)	NA
	Sbrana et al. [55]	Chronic lymphocytic leukaemia	Cell inclusion into a hydrogel matrix mixed with commercial bioinks Construction of 5 × 5 × 1 mm^3^ scaffolds with a bioprinter, using 50 × 10^6^ purified CLL cells per scaffold. No exogenous stimuli. Culture over 28 days.	*n* = 26	Viability of 66% at J14, 47% at d28.	NA	Higher viability at all time points in 3D compared to 2D (grown on laminin coated culture plates). Increased expression of anti-apoptotic and decreased expression of pro-apoptotic proteins in 3D compared to 2D.
Inclusion of tissue fragments	Li et al. [60]	Primary central nervous system lymphoma	Tumour sections are fragmented into pieces of 1–3 mm in diameter, placed within an incision into hPSC-derived brain organoids with matrigel. Culture over 20 days.	*n *= 9	8/9 samples yielded proliferating organoid culture.	Sensitivity to methotrexate and ibrutinib, no effect with dexamethasone and rituximab (*n* = 3 organoids).	NA
	Santamaria-Martinez et al. [58]	Various entities of B-cell and T-cell lymphomas	Tissue fragments of 0.75–1.5 mm^3^ are placed in inserts containing hydrogel with permeable bottom (air-liquide interface) Cytokine: BAFF. Culture over 3–7 days.	*n* = 27	No raw data regarding viability.	Exposure to small molecule inhibitors (ibrutinib, idelalisib, lenalidomide, venetoclax, tazemetostat, alisertib, everolimus, panobinostat, 5-azacytidine; *n* = 19 samples). Prediction of patient’s clinical response for 7 patients.	NA
Hard porous scaffolds	Barbaglio et al. [68]	Chronic lymphocytic leukaemia	Spongostan scaffolds populated with 2 × 10^5^ stromal cell line HS-5 and 3 × 10^6^ primary CLL cells from PB (15:1 ratio) in a rotating bioreactor. Culture over 3 days.	*n* = 6	No raw data regarding viability. Stromal cells retain CLL cells within the matrix.	Ibrutinib mobilised CLL cells out of the scaffold.	NA
	Belloni et al. [71]	Chronic lymphocytic leukaemia	Spongostan scaffolds populated with 2×10^5^ human lymphatic fibroblasts and human umbilical vein endothelial cells (1:1 ratio) and 2 × 10^6^ PBMC (10:1 ratio) in a rotating bioreactor. Culture over 5 days.	*n* = 49	Viability of approx. 90% at d5.	Ibrutinib mobilised CLL cells out of the scaffold. Venetoclax showed a slightly more pronounced effect in the BM compared to the LN 3D scaffolds.	Increased ECM production in 3D. Slightly higher viability in 3D compared to 2D. No difference upon treatment with venetoclax

Ratios are expressed as [tumour cells: other cells]. When comparing 3D and 2D cultures, identical conditions were maintained regarding stromal cells, immune cells, and cytokines, except for the addition of Matrigel or collagen, which were specifically used in the 3D models.

*ADSC* adipocyte-derived stem cells differentiated into lymphoid-like fibroblasts, *BM* bone marrow, *CLL* chronic lymphocytic leukaemia, *ECM* extracellular matrix, *FL* follicular lymphoma, *FRC* fibroblastic reticular cells, *LN* lymph node, *MCL* mantle cell lymphoma, *MDM* monocyte-derived macrophages, *NA* not assessed, *PB* peripheral blood, *PBMC* peripheral blood mononuclear cells, *PDX* patient-derived xenograft.

^a^The number of primary samples or replicates is equivocal in the original article.

### Hydrogel-based scaffolds

Moving to scaffold-based approaches, several models rely on embedding cells within a hydrogel matrix, which is then crosslinked to form defined structures. These structures can be shaped either by casting the cell–hydrogel suspension into moulds or by using bioprinting technologies. Hydrogels are crosslinked polymer networks with high water content that can emulate the mechanical properties of soft tissues, thereby supporting structural organisation, cellular activities, and potentially mimicking GC dynamics.

Foxall et al. [5] adapted the hanging drop method to form spheroids with cells embedded in a collagen matrix. They optimised the culture of primary lymphoma cells from DLBCL patients by co-culturing them with fibroblasts derived from adipocyte-derived stem cells and monocyte-derived macrophages obtained from PBMC. However, there was no significant difference in cell viability between 3D and parallel 2D co-culture at the same time point. Although treatment with rituximab induced a downregulation of CD20 and FcγRIIB on lymphoma cells in spheroids, it did not induce phagocytosis nor cell death, in contrast to the 2D condition. Of note, collagen concentration influenced drug diffusion within spheroids, with lower-density matrices (e.g. 1 mg/mL collagen versus 2 mg/mL) allowing better drug penetration.

Recently, a similar model investigated the role of the lymph node microenvironment in CLL [43]. Using primary CLL cells embedded in a type I collagen matrix to generate spheroids, Lenti et al. demonstrated that co-culture with fibroblastic reticular cells protected tumour cells from apoptosis. Treatment with ibrutinib or venetoclax resulted in higher apoptosis rates in 2D cultures than in 3D spheroids, and these rates were further reduced in the presence of stromal cells, highlighting the model’s ability to mimic the protective effects of the lymph node microenvironment

A synthetic so-called ‘organoid’ model has been engineered to recapitulate GC and plasma cell responses, first within a reactive context. Based on maleimide end-functionalized polyethylene glycol (PEG-MAL), the hydrogel is functionalized with integrin-binding peptides such as REDV and RGD, mimicking ECM components (vascular cell adhesion protein 1 and vitronectin, respectively) [44, 45] (Fig. 1d). The macromers are cross-linked with protease-degradable and non-degradable crosslinkers, allowing primary B-cell survival, as activated B cells and lymphoma cells express matrix metalloproteinases that participates in matrix remodelling in vivo [45, 46]. These hydrogels allowed co-culture of murine [44, 45] or human [47] naïve B cells with stromal cells (genetically engineered murine fibroblasts) expressing CD40L and secreting BAFF, promoting GC-like differentiation [44, 47]. Interestingly, these hydrogel-based immune tissues demonstrated more sustained GC and plasma cell responses when derived from peripheral blood mononuclear cells compared to tonsil-derived ones [48]. This bioengineered model has been also used to study lymphoma therapy responses, depending on TME interactions [46, 49]. Two studies demonstrated that co-culturing transformed FL (DOHH2) or DLBCL cell lines (HBL-1, SUDHL-6, OCY-LY10 and OCY-LY3) with stromal cells increased resistance to chemotherapies like doxorubicin, the histone deacetylase inhibitor panobinostat and inhibitors of the mucosa-associated lymphoid tissue lymphoma translocation protein 1 (MALT1) [46, 49]. Recently, primary cells isolated from patients with activated-B-cell (ABC)-type of DLBCL, xenografted into immunodeficient mice, and subsequently cultured within the organoid model in the presence of CD40L-expressing stromal cells, exhibited resistance to the MALT1 protein inhibitors [46]. However, in the absence of CD40L signalling, MALT1 inhibitors induced significant cell death, highlighting CD40L’s protective role. Beyond the involvement of supporting cells and CD40L signalling, the interaction between tumour cells and ECM proteins through integrins also contributes to chemoresistance [50]. Pharmacological inhibition of integrins using cilengitide, a selective inhibitor of αvβ3 and αvβ5 integrins, reduced tumour proliferation both in vitro and in xenograft models, demonstrating the therapeutic potential of targeting integrins [51]. Combination therapies targeting multiple microenvironmental pathways also represent promising therapeutic options to overcome resistance. For example, combining the MALT1 inhibitor MI2 with idelalisib—inhibitor of the phosphoinositide 3-kinase—decreased survival in both HBL-1 cell line and primary cells derived from patient-derived xenograft. However, primary cells obtained from patient-derived xenograft may not fully replicate the tumour’s heterogeneity due to biases introduced by repeated passages in mice, potentially skewing treatment response assessments.

In Hodgkin lymphoma (HL), Bahlmann et al. developed a biomimetic cryogel made of hyaluronan, gelatin, and fibronectin-derived peptides to study macrophage invasion [52, 53]. The Hodgkin-derived L428 cells promoted primary human macrophage invasion into the engineered cryogels, a phenomenon that was associated with a unique polarisation phenotype. In contrast, tumour-associated macrophages (TAM) cultured with conditioned medium from six non-Hodgkin lymphoma cell lines showed lower colonisation, highlighting the distinct behaviours of these lymphoma subtypes. The study also tested 25 therapeutic agents more likely to affect HL-TAM communication. Five compounds were identified that reduced TAM colonisation. Notably, the p38 mitogen-activated protein kinase inhibitor re-polarised macrophages into a pro-inflammatory phenotype, while the inhibitors of the signal transducer and activator of transcription STAT6 and the Janus kinases ½ reduced the activity of pro-inflammatory genes. Of particular interest, ruxolitinib is currently being investigated in phase II clinical trials for HL [54]. This platform allowed the modelling of macrophage invasion in HL and identified potential macrophage-targeting therapeutics.

More recent advances in 3D bioprinting have enabled further development of hydrogel-based scaffolds. 3D bioprinting is an additive manufacturing technique in which bioinks are constituted of cells resuspended in biocompatible materials, and are deposited in a layer-by-layer process to a previously defined geometry. This allows the generation of multiple scaffolds with high levels of reproducibility, enabling precise control of scaffold composition and specific architecture of the structure. Sbrana et al. [55] reported the use of this emerging technology in CLL. Primary CLL cells and the MEC1 cell line were embedded in commercial bioinks and bioprinted using a pneumatic extrusion printer into 5 × 5 × 1 mm³ lattice-like constructs. Constructs were then cultured in appropriate media under standard conditions. Mechanical properties of cell-laden and acellular gels were characterised by compression testing, revealing stiffness comparable to lymphoid tissues. Cell viability and spatial distribution within the scaffold were monitored up to 28 days, showing markedly improved survival of CLL cells in 3D compared with 2D cultures on laminin-based substrates, without the addition of stromal cells or exogenous stimuli in both conditions.

In conclusion, hydrogel-based models represent efficient tools to recapitulate cellular cues, ECM components and mechanical properties within the GC context. They offer the advantage of tunable mechanical stiffness, which likely modulates the growth of B-cell lymphomas [56]. Bioprinting technologies could further enhance the complexity of these approaches.

### Inclusion of primary tissue fragments into supportive matrices

Santamaria-Martinez et al. adapted the air-liquid interface method [57] to generate patient-derived tumoroids referred to as ‘lymphomoids’ [58] (Fig. 1e). Tissue fragments from fresh biopsies were embedded in an RGD-based hydrogel supplemented with BAFF and could be maintained for up to 7 days while preserving the cellular heterogeneity and spatial organisation of the original tissue, as assessed by flow cytometry, single-cell RNA sequencing and spatial transcriptomics analyses. Twenty-seven samples from patients with various lymphoma subtypes were successfully cultured, including ten DLBCL, five primary mediastinal B-cell lymphomas, two high-grade B-cell lymphomas, two small lymphocytic lymphomas, one MCL, one marginal zone lymphoma, three FL, one transformed FL, one Burkitt lymphoma, and one T-cell lymphoma. Of these, 19 samples were subjected to drug screening using a panel of small molecule inhibitors to evaluate patient-specific tumour sensitivity. In seven cases, patients received one of the tested compounds, and clinical response data were available, showing that the in vitro sensitivity profiles of the lymphomoids mirrored the patients’ actual treatment responses.

Recently, Li et al. leveraged existing technologies in brain organoids [59] to generate a 3D model specific of primary cerebral nervous system lymphoma (PCNSL) [60]. By inserting fragmented tumour tissue samples into wild-type brain organoids derived from pluripotent stem cells (Fig. 1f), they successfully created and maintained PCNSL organoids from 8 patients over a 14-day culture period. An in vivo imaging system was used to monitor the signal intensity and size of the PCNSL organoids, which had been transfected with luciferase-labelled adenovirus. The PCNSL organoids exhibited a high similarity to their parental tumours, as assessed by comparing copy number variations, single-nucleotide variations, and gene expression profiling. The responses of the PCNSL organoids to chemotherapeutic and targeted drugs were evaluated, showing that they could serve as a valuable tool for therapeutic screening.

This approach offers the clear advantage of faithfully representing the original tumour; however, the range of experiments feasible with this model is limited by the quantity of primary tissue available.

### Organ-on-chip models

Organ-on-chip models are microfluidic devices enabling culture of live cells within micrometre-sized chambers that are continuously perfused [61,62]. Existing systems range from simple chambers with a single cell type to more complex configurations with multiple chambers and cell types to recreate tissue interactions. This technology allows precise control of various parameters through the creation of microchannels in complex 3D culture systems.

A DLBCL-specific organ-on-chip model features a microfluidic structure with a hydrogel reservoir designed to mimic the TME [63]. The hydrogel, formed from Glycosil and Gelin-S, is embedded within a polydimethylsiloxane macrostructure and contains murine A20 tumour cells (a cell line equivalent to human DLBCL), CD3^+^ T cells, and CD11b^+^ macrophages. A microchannel, created by removing a stainless-steel wire after hydrogel polymerisation, allows the introduction of pulmonary microvascular endothelial cells via perfusion, simulating a perfusable blood vessel to model interactions between tumour and endothelial cells [63] (Fig. 1g). This system showed that antibodies targeting the macrophage colony-stimulating factor 1 receptor (CSF1R) selectively induced macrophage apoptosis when perfused through the microchannel [63]. Interestingly, antibody diffusion was enhanced in the presence of tumour cells, aligning with in vivo findings of increased endothelial permeability in DLBCL [63, 64]. Although this model uses murine cells rather than primary DLBCL cells, it offers a robust platform for analysing targeted therapies and tumour-endothelial interactions.

Mastini et al. [65] used a 3D microfluidic chip model to simulate the perivascular niche in anaplastic large cell lymphoma (ALCL). They used a commercial microfluidic chip ‘3-D cell culture chip’ (DAX-1, AIM Biotech), which consists of a central collagen hydrogel channel representing the perivascular niche, flanked by two lateral channels mimicking blood vessels (Fig. 1h). To create the 3D structure, collagen hydrogel was injected into the central region. The walls of lateral channels were coated with collagen to improve adhesion of human umbilical vein endothelial cells that formed a monolayer at the media-gel interface, creating the 3D macrovessel. The following day, lymphoma cells (ALCL cell lines KARPAS-299, DEL and COST) were introduced within the perfusable macrovessel, interacting with endothelial cells. In this model, the presence of endothelial cells that produce CCL19 and CCL21 protected ALCL cells from the apoptotic effects induced by crizotinib and through the engagement of the CCR7 receptor. This work confirmed the critical role of the TME in supporting the persistence of large cells anaplastic lymphoma cells after chemotherapy.

Therefore, organ-on-chip models offer valuable tools for replicating aspects of the TME, with the DLBCL and the ALCL studies focusing on endothelial-tumour interactions. Both systems revealed critical insights into tumour biology and drug responses. Given their ability for large-scale production and reproducibility, organ-on-chip platforms have the potential for high-throughput drug screening in preclinical research, but their high cost and specialised manufacturing requirements limit widespread use.

### Hard material-based, preformed scaffolds

Besides cell inclusion in hydrogels, the other main technique for developing 3D scaffold-based models involves seeding cells into a preformed scaffold that can be polymeric porous (either synthetic or natural) materials, or a decellularized human scaffold. These platforms are designed to accurately represent the geometric structure and spatial arrangement of cells specific to a given tissue. Specifically, recent studies explored the innovative use of bone scaffolds or actual bone as moulds to create biologically relevant simulations of the BM environment. While BM involvement is common in CLL, it is less frequent in lymphomas and is associated with a poor prognosis. These particular cases necessitate specialised 3D models, as the BM provides a specific ‘niche’ that includes cellular components such as osteoblasts, endothelial cells, and mesenchymal stem cells (MSC), along with non-cellular elements like the ECM and signalling molecules.

#### Synthetic hard porous scaffolds

Caicedo-Carvajal et al. [66] used an original 3D polystyrene scaffold to study the influence of stromal environment on the growth of MCL cell lines. The scaffold is made of four layers of polymers with controlled pore size and porosity, and seeded with a mixture of MCL cells (HBL-2 or Z-138 cell lines) and human dermal fibroblasts (Fig. 1I). The co-culture caused the HBL-2 cells to aggregate in clusters, whereas they grew mostly as single-cell suspension in the absence of stromal cells. Proliferation and cluster formation was increased in the 3D scaffold compared to the 2D condition. However, the growth dynamics varied depending on the tumour origin, with lymph node-derived HBL-2 cells forming robust clusters, whereas BM-derived Z-138 cells showed limited proliferation, even in the presence of stromal cells, highlighting the critical need to provide niche-specific signals to the studied tumour to develop a relevant model.

#### Decellularized human scaffolds

As BM involvement confers a poor prognosis at DLBCL diagnosis, Ceccato et al. developed a 3D model based on a human bone scaffold to study the growth and drug resistance of DLBCL lymphoma cells within the native biochemical and biophysical characteristics of the BM compartment [67]. Human femoral bone fragments were decellularized and recellularized with primary BM-MSC, then incubated with DLBCL cell lines (Fig. 1j). The lymphoma cells were able to migrate, adhere, and expand both in the decellularized and BM-MSC recellularized scaffolds. They maintained the expression of CD19, CD20, and CD45 over time, but they adopted a pseudopod-like shape, suggesting that the interaction with the stroma triggered cytoskeletal organisation. GC-derived OCI-LY18 cells were more resistant to doxorubicin-induced apoptosis when growing in the decellularized 3D bone scaffold compared to 2D cultures, suggesting a protective role of the ECM. However, the coexistence of MSC in the 3D scaffold did not significantly affect doxorubicin-induced apoptosis. In contrast, ECM did not protect the ABC-derived NU-DUL-1 lymphoma cell line from doxorubicin-induced apoptosis, but protection was observed when MSC were growing in the bone scaffold. These results suggest that the interaction of lymphoma cells with the microenvironment may differ according to the DLBCL subtype and that 2D systems may fail to reveal this behaviour. However, this model has not been tested with primary DLBCL cells.

#### Collagen-based hard porous scaffolds

Barbaglio et al. [68] proposed a scaffold-based 3D model of the BM microenvironment using Spongostan^TM^, a collagen sponge with an ultrastructure similar to the trabecular structure of BM. This model, previously validated for multiple myeloma, was adapted to study the homing and migration of CLL cells in response to treatment. Spongostan^TM^ scaffolds were sequentially populated with the human BM-derived stromal cell line HS5 and either the CLL cell line MEC1 or primary CLL cells. The scaffolds were then transferred to High Aspect Ratio Vessels, which allow oxygen and nutrients diffusion through an exchange membrane, and cultured in a rotating bioreactor (Fig. 1k). This setup recreates BM-like conditions and enables interactions between CLL cells and BM stromal cells. Parallel analyses of cells inside and outside the scaffold revealed that stromal cells were necessary to efficiently retain CLL cells within the matrix. Moreover, leukaemic cells with reduced expression of the hematopoietic cell-specific Lyn substrate-1 (HS1) protein preferentially remained within the biomaterial, mirroring the lower HS1 expression observed in BM-derived CLL cells compared to those from peripheral blood. Treatment with ibrutinib mobilised CLL cells out of the scaffold; however, cells with inactive HS1 were mobilised less efficiently than those with active HS1. These findings suggest a role for HS1 in the compartmentalisation of CLL cells and underscore its potential link to aggressive disease prognosis, as BM-sequestered CLL cells – protected by their microenvironment – often display reduced sensitivity to anticancer therapies [69, 70].

The same group further adapted this model to mimic a three-dimensional lymph node structure. In this system, a Spongostan-based scaffold was seeded with human lymphatic fibroblasts and endothelial cells, which deposited extracellular matrix and supported the viability and proliferation of patient-derived CLL cells [71]. Treatment with venetoclax revealed stronger protection of CLL cells within the lymph node–like environment than in the bone marrow, whereas the mobilising effect of ibrutinib was comparable in both settings.

Finally, the model was further enhanced by connecting the bioreactors containing the BM and lymph node scaffolds to a peristaltic pump to ensure continuous medium perfusion through the scaffolds, with specific flow rates applied to each compartment to simulate the fluid dynamics of in vivo tissues [72]. Systematic comparisons with the same system under static conditions revealed that dynamic flow promoted more physiological cell morphology, enhanced ECM maturation, increased cell-cell interactions, and more homogeneous cell distribution within the scaffolds. Thus, dynamic stimulation—providing convective transport of oxygen, nutrients, and growth factors while replicating in vivo mechanical forces—enables more accurate tissue-like organisation in both BM and lymph node constructs. This seminal study, although restricted to the use of a CLL cell line, demonstrated that not only 3D architecture but also dynamic stimulation critically influences cell behaviour, offering crucial insights for studying the complex pathophysiology of haematological malignancies.

## Conclusions

3D platforms have proven valuable for studying CLL and lymphomas by partially recapitulating tumour complexity, particularly through the integration of key TME components. Numerous studies demonstrate that cells cultured in 3D exhibit distinct behaviours compared to 2D cultures, more closely mimicking in vivo conditions and enabling complex intercellular communication as well as cell-ECM interactions. While these advantages have spurred widespread adoption, most 3D models employ scaffold-free approaches, valued for their low cost and straightforward implementation in standard laboratories. Nevertheless, these models exhibit limited complexity and fidelity in replicating the intricate 3D architecture of lymphoid organs, TME diversity, or the dynamic nature of haematological malignancies. Consequently, more sophisticated scaffold-based and dynamic models have been developed to overcome these limitations and better recapitulate the structural and functional complexity of lymphoid tissues.

Bioprinting is a groundbreaking technology that incorporates different types of tumour and non-tumour cells into a bioink, such as stromal, endothelial, and immune cells, enabling increased model complexity. In addition to reproducing the cellular heterogeneity of tumours, it offers precise control over construct architecture, including the compartmentalisation of specific niches, and thus provides a more realistic representation of the 3D TME. Despite these advantages, 3D bioprinting also has limitations related to the resolution and precision of the printed structures, which often lack the fine detail of native tissues. Most importantly, the high cost of this technology remains a major limiting factor [73] and likely contributes to the low number of studies reporting its use in haematological malignancies [55, 74, 75].

Besides the 3D architecture and TME composition, numerous biophysical forces shape hematopoietic organs, notably pressure and shear stress generated by drag forces from blood flow, lymphatic circulation, or interstitial flow on vessel walls and within tissues [76–78]. Physiological shear stress on lymphatic endothelial cells upregulates adhesion molecules, optimising immune cell entry and fluid transport into lymph nodes while strengthening endothelial barrier function through cytoskeletal remodelling [79–81]. Computational models further reveal spatially heterogeneous shear patterns in the subcapsular sinus and lymphoid compartment, influencing cell adhesion and migration [72, 82]. Like healthy leucocytes, tumour lymphoid cells sense both biochemical and mechanical cues from their surrounding tissues and respond to bloodstream shear stress, which triggers cytoskeletal reorganisation and deformation essential for endothelial transmigration [83, 84]. To recapitulate these biomechanical cues, advanced 3D models—such as organ-on-chip and microfluidic platforms—have emerged to integrate physiological shear stress into lymphoid tissue mimics [85]. These systems replicate laminar and pulsatile flow through endothelialized channels lined with lymphatic endothelium or high endothelial venules, enabling real-time imaging of immune cell diapedesis and stromal interactions under controlled fluid dynamics [63, 65]. Dynamic bioreactors with perfused flow, as developed for CLL models [72, 84], also represent promising approaches to recreate the journey of blood cells within and between specific hematopoietic compartments. Although expensive and technically demanding, the ability of such platforms to impose gradient-driven shear and cyclic strain positions them as critical tools for investigating mechanosensitive tumour-stroma crosstalk and drug penetration under flowing conditions. Ultimately, these complex systems could pave the way for perfused multi-organ platforms interconnecting multiple compartments. Such multi-tissue integrated constructs would incorporate diverse tissue microenvironments, each with its specific biochemical and mechanical cues.

Beyond the use of 3D models for preclinical drug testing, predicting a patient’s individual response to chemotherapy remains challenging. Despite major advances in the development of targeted therapies (such as ibrutinib, venetoclax, and tazemetostat) over the past few years, clear guidelines for their personalised use are still lacking. Therefore, evaluating the potential of 3D models to predict patient’s individual response to drugs will constitute a key objective in future studies. Moreover, the successful long-term maintenance of primary lymphoma cells remains heterogeneous across studies. It will be of utmost importance that the systems can maintain viability of lymphoma cells in vitro over several days, allowing testing of long-acting therapies such as epigenetic agents.

Thus, promising models have emerged, yet ongoing efforts are essential to optimise the representativeness of the in vivo dynamic reactions. The development of platforms that enable reproducible, large-scale production for high-throughput drug screening will become indispensable for advancing next-generation personalised medicine. These advancements will provide a better understanding of tumour biology, guide drug development, and improve clinical decision-making.
